# High-Fidelity simulation-based program improves flow state scale in the perinatal team

**DOI:** 10.1186/s13052-021-00972-2

**Published:** 2021-02-25

**Authors:** Mariachiara Martina Strozzi, Alessandro Varrica, Micaela Colivicchi, Claudia Pelazzo, Rossana Negri, Anna Galante, Patrizia Ianniello, Rossella Sterpone, Priscilla Nannini, Daniela Leo, Francesca Mannarino, Manuel Striani, Stefania Montani, Diego Gazzolo

**Affiliations:** 1 Neonatal Intensive Care Unit AO S.S. Antonio, Biagio and C. Arrigo Hospital, Alessandria, Italy; 2 Department of Paediatric Cardiovascular Surgery, Laboratory Research, San Donato Milanese University Hospital, San Donato, Italy; 3 Psychology Unit AO S.S. Antonio, Biagio and C. Arrigo Hospital, Alessandria, Italy; 4 Science and Technological Innovation Department, University of Piemonte Orientale, Alessandria, Italy; 5 Neonatal Intensive Care Unit G. D’Annunzio University of Chieti, Chieti, Italy; 6 Department of Maternal, Fetal and Neonatal Medicine, C. Arrigo Children’s Hospital, I-15100 Alessandria, Italy

**Keywords:** Simulation based training, Delivery room, Neonatal resuscitation, Flow state scale test

## Abstract

**Background:**

We aimed to evaluate the degree of realism and involvement, stress management and awareness of performance improvement in practitioners taking part in high fidelity simulation (HFS) training program for delivery room (DR) management, by means of a self-report test such as flow state scale (FSS).

**Methods:**

This is an observational pretest-test study. Between March 2016 and May 2019, fourty-three practitioners (physicians, midwives, nurses) grouped in multidisciplinary teams were admitted to our training High Fidelity Simulation center. In a time-period of 1 month, practitioners attended two HFS courses (model 1, 2) focusing on DR management and resuscitation maneuvers. FSS test was administred at the end of M1 and M2 course, respectively.

**Results:**

FSS scale items such as unambiguous feed-back, loss of self consciousness and loss of time reality, merging of action and awareness significantly improved (*P* < 0.05, for all) between M1 and M2.

**Conclusions:**

The present results showing the high level of practitioner involvement during DR management-based HFS courses support the usefulness of HFS as a trustworthy tool for improving the awareness of practitioner performances and feed-back. The data open the way to the usefulness of FSS as a trustworthy tool for the evaluation of the efficacy of training programs in a multidisciplinary team.

## Introduction

Epidemiological data reported that, at birth, about 10% of newborns respond during drying and stimulation maneuvers, approximately 3% initiate respiration after positive-pressure ventilation (PPV), 2% need intubation to support respiratory function and 0.1% require chest compressions and/or epinephrine to achieve transitional phase [1–4]. Therefore, delivery room (DR) management still constitutes a huge challenge for the neonatal team. In this regard, a multidisciplinary approach based on technological, diagnostic, therapeutic and, last but not least, technical (TS) and non-technical skills (NTS) training has been shown to improve DR management [4–8]. NTS, such as team coordination, decision-making, situation awareness and communication, have been previously defined as the cognitive and interpersonal skills that complement an individual’s professional and technical knowledge in the facilitation of effective delivery of a safe healthcare system [8–12]. NTS can be implemented through High Fidelity Simulation (HFS)-based training and Crisis Resource Management (CRM) training modules. HFS is an educational traning methology characterized by equipment, environmental and psychological high fidelity, the last of which is generally considered the most important for training and learning [13–18].

In the last decade, HFS has been increasingly adopted for clinical training across the medical education continuum [18–25]. The 2015 International Neonatal Resuscitation Guidelines supported HFS as a standard and essential component in neonatal resuscitation training [2–4, 7, 13], including training practitioners for stress during DR procedures [25, 26]. Thus, the use of stress and motivation evaluation tests such as the Flow State Scale (FSS) could be of help [25–30]. FFS consists of 36 items divided into 9 subscales, each of which representing a different dimension [31–33]. It is a powerful motivational tool and it is related to skills development [34–41]. At this stage, data evaluating FFS changes during team training in HFS is still lacking.

The purpose of the present study was to investigate study practitioners’ flow experiences during in-house HFS courses characterized by progressive complexity. Our hypothesis was that training with HFS would improve the experience of realism for trainees, thus improving motivation and reducing stress in successive training.

## Methods

Between March 2016 and May 2019 we conducted an observational pretest-test study on 43 practitioners admitted to our training center. The characteristics of the recruited subjects are reported in Table 1.

**Table 1:** General characteristics of practitioners admitted in the study

Parameter	Data	N°	Percent
Gender	Female	40	93
	Male	3	7
Work Role	Nurse	25	58
	Midwife	7	16
	Anesthetist	2	5
	Pediatrician	8	19
	Gynecologist	1	2
Seniority work	< 5	11	26
	5–10	9	20
	10–20	12	28
	> 20	11	26
Hospital’s Level	II	17	39.5
	III	26	60.5
Previous Course	Yes	28	65
	No	15	35
Previous HFS Course	Yes	0	0
	No	43	100

The Neonatal Intensive Care Unit of C. Arrigo Children’s Hospital, Alessandria, Italy is the III level Piedmont regional referral centre for neonatal intensive care and for training in neonatal emergency assessment. The neonatal HFS centre of Alessandria has been operating since 2012 within the hub and spoke welfare network. The center also offers HFS courses to other extraregional practitioners.

### Neonatal high Fidelity simulation center

The neonatal HFS centre at C. Arrigo Children’s Hospital consists of a scenario room with DR or neonatal intensive care bed, a director’s room and classrooms for theoretical lessons and debriefing. The simulation room was modified specifically to have the appearance of a real DR or neonatal intensive care bed. Participants had all the necessary materials for attending to a newborn available, according to the latest American Heart Association (AHA) and Academy of Pediatrics (AAP) recommendations, including: an oxygen-air blender; a T-piece resuscitator (Neo-Tee® Infant T-Piece Resuscitator Mercury Medical, Clearwater, Florida, USA); respiratory support devices for invasive (Leoni Plus, Heinen Lowenstein, Rheinland-Pfalz, Germany) and non invasive (Intant Flow, CareFusion, Hoechberg, Germany) ventilation [2–5].

During scenario performance it was possibile to see the patient’s vital signs and laboratory or instrumental tests on a specific monitor. Scenarios were performed by using newborn simulators (SimNewB and Premature Anne, Laerdal Medical Corporation, Laerdal, Stavanger, Norway). SimNewB is highly realistic neonatal simulator with one size and weight of a newborn baby girl delivered at term with approximately 3.5 Kg body weight. Premature Anne is a highly realistic 25 week preterm infant simulator with an approximate weight of 0.6 Kg.

A recording system with three high-definition cameras and an ambient microphone located in the resuscitation warmer was used.

### HFS courses

The HFS courses were performed in a time period of 1 month and sub-grouped into two separate sections including theoretical and videos lessons, TS exercises, scenario performances, de-briefings and psychological tests. At the beginning of each section practitioners were grouped into multidisciplinary teams of 3–4 persons (obstetrician, neonatologist, midwife, pediatric nurse) and underwent simulator suite orientation (familiarization).

Sections were characterized by progressive complexity in training, as follows:

### Model 1 (M1)

**Fig. 1:**
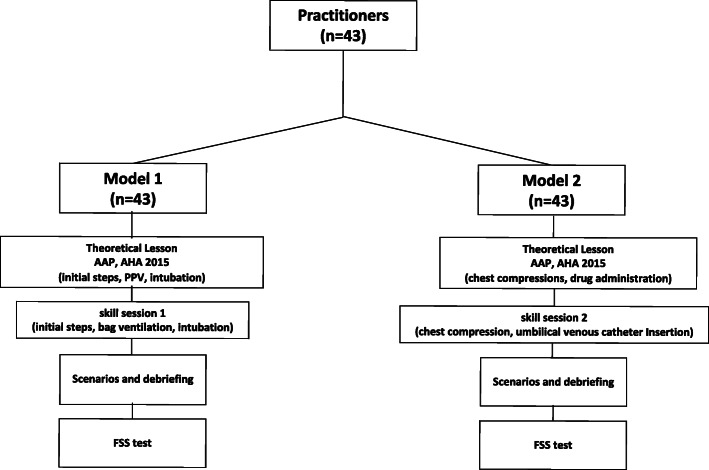
Flow-chart of the training program according to the American Academy of Pediatrics (AAP) and American Heart Association (AHA) neonatal resuscitaton statements. Flow State Scale (FSS) test was administrated to practiotioners at the completion of every training model

The M1 consisted of a 1-day training course (Fig. 1) with theoretical lessons and videos on the following themes: first minute International Newborn Resuscitation Guidelines, AHA and AAP recommendations [2–5]; human error and teamwork simulation-based learning in neonatal resuscitation; NTS; teamwork and European Resuscitation Council (ERC) recommendations for structured multidisciplinary communication. After the theoretical session, practitioners underwent a TS session regarding invasive and non-invasive (stimulation, PPV, intubation) DR resuscitation maneuvers.

TS session was characterized by 3 standardized code scenarios included in the Neonatal Resuscitation Program (NRP) regarding DR resuscitation critical situations. Scenarios were in accordance with standardized models provided by NRP. During practical performance the remaining multidisciplinary teams were able to evaluate the TS procedures via a video-live connection. After practical activities practitioners participated in an video-interactive debriefing session.

### Model 2 (M2)

The M2 consisted of a 1-day training course (Fig. 1) conducted 15 days after M1 with theoretical lessons and videos about the following themes: starting from the second minute International Newborn Resuscitation Guidelines, AHA and AAP recommendations [2–5]; human error and teamwork simulation-based learning in neonatal resuscitation; NTS; teamwork and ERC recommendations for structured multidisciplinary communication. After the theoretical session, practitioners took part in a TS session regarding non-invasive and invasive (chest compressions, drug administration) DR resuscitation maneuvers.

TS session was characterized by 3 standardized code scenarios included in the NRP regarding both 1^st^ and 2^nd^ minute DR resuscitation situations. Scenarios were in accordance with standardized models provided by NRP. During practical performance the other multidisciplinary teams were able to evaluate the TS procedures via a video-live connection. After practical activities, practitioners participated in an interactive video-debriefing session where they can watch themselves during the debriefing session and evaluate, together with the teaching team, the quality of their performance.

The teaching team was formed of 8 expert trainers (neonatologists: *n* = 4; pediatric nurses: n = 4) in simulation, debriefing, teamwork and communication, certified by the Italian Society of Neonatology (SIN).

### Flow state scale

The FSS test was administered to practitioners at the end of M1 and M2 scenario, respectively. The FSS consists of 36 items divided into 9 subscales, the short flow scale (SFSS), each representing the nine different dimensions of Flow (Tables 2, 3). These nine dimensions are: challenge-skill balance, action-awareness merging, clear goals, unambiguous feedback, concentration on task, sense of control, loss of self-consciousness, time transformation, and autotelic experience. Considered together, these dimensions represent the optimal psychological state of flow; singly they signify conceptual elements of this state (Table 3). The 36 items (Pi) also contribute to a broad second-order general flow factor (Table 2). Participants were asked to answer all 36 items after scenario session. Each item was scored from 1 to 5 as follows: never, 1; rarely, 2; sometimes, 3; frequently, 4; always 5.

**Table 2:** Flow state scale test items (Pi) results recorded at the end of Model 1 (M1) and Model 2 (M2) high fidelity simulation scenarios in the 43 trainees admitted in the study

Pi	Description	M1 (***n*** = 43)			M2 (***n*** = 43)			P
		Median	25° centile	75° centile	Median	25° centile	75° centile	
1	I am challenged, but I believe my skills will allow me to meet the challenge	3.00	3.00	3.00	3.00	3.00	3.00	0,928
2	make the correct movements without thinking about trying to do so	3.00	2.00	3.00	3.00	2,00	3.00	0,111
3	I know clearly what I want to do	3.00	3.00	4.00	3.00	3,00	4.00	0,832
4	**It is really clear to me how my performance is going**	3.00	2.00	3.00	3.00	3,00	3.75	***0,038***
5	My attention is focused entirely on what I am doing	4.00	3.00	3.00	4.00	4.00	5.00	0,060
6	I have a sense of control over what I am doing	3.00	3.00	3.75	3.00	3.00	3.00	0,444
7	**I am not concerned with what others may be thinking of me**	3.00	2.00	3.00	3.00	2.00	4.00	***0,007***
8	Time seems to alter (either slows down or speeds up)	4.00	2.00	4.00	3.00	2.25	4.00	0,789
9	I really enjoy the experience	4.00	3.00	4.00	4.00	3.00	4.00	0,522
10	My abilities match the high challenge of the situation	3.00	3.00	3.00	3.00	3.00	4.00	0,330
11	Things just seem to happen automatically	3.00	3.00	3.00	3.00	3.00	4.00	0,051
12	I have a strong sense of what I want to do	3.00	3.00	3.00	4.00	3.00	4.00	0,270
**13**	**I am aware of how well I am performing**	**3.00**	**2.00**	**3.00**	**3.00**	**2.25**	**3.00**	***0,039***
14	It is no effort to keep my mind on what is happening	3.00	3.00	4.00	4.00	3.00	4.00	0,057
15	I feel like I can control what I am doing	3.00	3.00	3.00	3.00	3.00	3.00	0,146
16	I am not concerned with how others may be evaluating me	3.00	2.00	3.00	3.00	3.00	4.00	0,058
17	The way time passes seems to be different from normal	4.00	3.00	4.00	3.00	3.00	4.00	0,736
18	I love the feeling of the performance and want to capture it again	3.00	3.00	4.00	3.00	3.00	4.00	0,802
19	I feel I am competent enough to meet the high demands of the situation	3.00	3.00	3.00	3.00	3.00	3.00	0,710
20	I perform automatically, without thinking too much	3.00	2.25	4.00	3.00	3.00	4.00	0,385
21	I know what I want to achieve	3.00	3.00	4.00	4.00	3.00	4.00	0,503
22	I have a good idea while I am performing about how well I am doing	3.00	3.00	3.00	3.00	3.00	3.00	0,610
23	I have total concentration	4.00	3.00	4.00	4.00	3.00	4.00	0,333
24	I have a feeling of total control	3.00	2.25	3.00	3.00	3.00	3.00	0,228
**25**	**I am not concerned with how I am presenting myself**	**3.00**	**2.00**	**3.00**	**3.00**	**2.25**	**4.00**	***0,014***
**26**	**It feels like time goes by quickly**	**3.00**	**1.25**	**4.00**	**3.00**	**2.00**	**4.00**	***0,042***
27	The experience leaves me feeling great	3.00	3.00	4.00	4.00	3.00	4.00	0,093
28	The challenge and my skills are at an equally high level	3.00	3.00	3.00	3.00	3.00	3.00	0,339
29	I do things spontaneously and automatically without having to think	3.00	2.25	3.00	3.00	3.00	4.00	0,148
30	My goals are clearly defined	4.00	3.00	4.00	4.00	3.00	4.00	0.836
31	I can tell by the way I am performing how well I am doing	3.00	2.00	3.00	3.00	3.00	3.00	0.170
32	I am completely focused on the task at hand	4.00	3.00	4.00	4.00	3.00	4.00	0.713
33	I feel in total control of my body	3.00	3.00	4.00	3.00	3.00	4.00	0.931
34	I am not worried about what others may be thinking of me	3.00	2.00	3.00	3.00	2.00	4.00	0.018
35	I lose my normal awareness of time	**3.00**	**2.00**	**3.00**	**3.00**	**2.25**	**4.00**	***0.029***
36	The experience is extremely rewarding	3.00	3.00	4.00	4.00	3.00	4.00	0.308

**Table 3:** Short Flow State Scale (SFSS) results recorded at the end of Model 1 (M1) and Model 2 (M2) high fidelity simulation scenarios in the 43 trainees admitted in the study

Short Flow Flow State Scale – Subscale dimension									
		Items	M1 (n = 43)			M2 (***n*** = 43)			P
SFSS			median	25°	75°	median	25°	75°	
1	Challenge-Skill Balance	Pi1 + Pi10 + Pi19 + Pi28	12.00	11.00	13.00	12,00	12.00	14.00	0.53
**2**	**Merging of Action and Awareness**	**Pi2 + Pi11 + Pi20 + Pi29**	**12.00**	**10.00**	**13.00**	**13.00**	**11.00**	**14.00**	***0.04***
3	Clear Goals	Pi3 + Pi12 + Pi21 + Pi30	13.00	12.00	15.00	14,00	12.25	15.00	0.37
**4**	**Unambiguous Feedback**	**Pi4 + Pi13 + Pi22 + Pi31**	**12.00**	**9.25**	**13.00**	**12.00**	**11.00**	**14.00**	***0.04***
5	Concentration on the Task at Hand	Pi5 + Pi14 + Pi23 + Pi32	15.00	13.00	16.00	15,00	14.00	17.00	0.06
6	Sense of Control	Pi6 + Pi15 + Pi24 + Pi33	12.00	11.00	13.00	12.00	12.00	14.00	0.13
**7**	**Loss of Self-Consciousness**	**Pi7 + Pi16 + Pi25 + Pi34**	**11.00**	**08.00**	**12.00**	**13.00**	**9.50**	**15.00**	***0.002***
8	Transformation of Time	Pi8 + Pi17 + Pi26 + Pi35	11.74	10.00	13.00	13.09	12.40	14.00	0.11
9	Autotelic Experience	Pi9 + Pi18 + Pi27 + Pi36	13.90	12.80	14.90	14.60	13.40	15.50	0.26

*Abbreviation*: flow state scale item (Pi)

The study protocol was approved by the local Ethics Committee (ASO.neonat.00022886) and the subjects examined gave informed and signed consent.

### Statistical analysis

For sample size calculation, we used changes in FFS as the main parameter [25]. We assumed an increase of 0.5 SD in FFS to be clinically significant. Considering an α = 0.05 and using a two-sided test, we estimated a power of 0.90, recruiting 41 practitioners. We added *n* = 2 cases to allow for dropouts and consent not given. The study population was therefore composed of 43 practitioners who underwent M1 and M2 training sections (Fig. 1). FFS values were expressed as median and 25-75^th^ centiles. Data were analyzed by Kruskal-Wallis one-way analysis of variance and Mann-Whitney U test when not normally distributed. Statistical significance was set at *P* < 0.05.

## Results

In Table 1 the characteristics of the practitioners admitted to the study are reported. Higher female (*P* < 0.001) incidence was observed when study group was corrected for gender. As expected, in term of the role of the recruited practitioners the incidence of nurses participating in M1 and M2 models was significantly (*P* < 0.05) higher than for other practitioners. Moreover, the number of obstetricians and anesthetists participating was significantly lower (*P* < 0.01) than for other disciplines (midwives, pediatricians/neonatologists, nurses). No significant differences (*P* > 0.05, for all) in terms of seniority were observed among different categories. Of note, the number of practitioners coming from I-II hospital levels was significantly lower (P < 0.05) than those coming from III level referral centres. Twenty-eight out of 43 practitioners had previously experienced resuscitation courses but none of them HFS courses.

In Table 2 the characteristics of the 36 FSS items are reported. No significant (P > 0.05, for all) statistical differences have been found between the two training sections regarding 29 out of 36 FFS items (Pi1-Pi3; Pi5, Pi6; Pi8–12; Pi14-Pi24; Pi27-Pi33; Pi36, respectively). Conversely, higher Pi4 (it was really clear to me how my performance was going), Pi7 (I was not concerned about what others might be thinking of me), Pi13 (I was aware of how well I was performing), Pi25 (I was not concerned about how I was presenting myself), Pi26 (it felt that time went by quickly), Pi34 (I was not worried about what others might be thinking of me) and, Pi35 (I lost my normal awareness of time) were observed in the studied group at the end of M2 performance.

In Table 3 the SFSS characteristics at M1 and M2 are reported. No significant differences have been found between M1 and M2 regarding SFSS1, SFSS5, SFSS6, SFSS8 and SFSS9, respectively. Conversely, higher (*P* < 0.05, for all) SFSS values at M2 than M1 were found regarding SFSS2 (merging of action and awareness), SFSS4 (unambiguous feed-back), and SFSS7 (loss of self-consciousness).

## Discussion

Approximately 10% of all newborns require resuscitation at birth [1–4]. Training healthcare providers in standardised formal neonatal resuscitation training programmes may improve neonatal outcomes [4–8]. However, despite a reduction of early neonatal and 28-day mortality further trials are required to enable a significant decline in the incidence of neonatal morbidity, including hypoxic ischaemic encephalopathy and neurodevelopmental outcomes. Therefore, innovative educational methods able to enhance knowledge, skills and teamwork behaviour are eagerly sought [4–8]. In this regard, there is growing evidence that DR management by a multidisciplinary team can be ameliorated by HFS team training [15–18]. HFS can improve knowledge, behaviour and practice through training in new techniques focused on experiential learning [16–18]. However, data on the effectiveness of HFS on stress-training of practitioners are still needed.

In the present study we found that in a cohort of practitioners taking part in HFS scenarios of progressive severity in DR resuscitation management, the administration of a well-established attitudinal test of stress-event management, namely FSS, showed significant improvement from M1 to M2 in 7 out of 36 FSS items. Furthermore, when FSS results were subgrouped into nine subscales expression of self-consciousness during emergency experience, a significant improvement was observed in three out of the nine analyzed.

The findings partly match previous observations in HFS pediatric programs. The discrepancy lies in terms of teaching program topics (pediatrics vs neonatologists) and scenarios (septic shock and severe asma vs DR resuscitation) [25]. Another explanation lies in the technical and timing of TS performances (2 sessions of 90 min vs one-day M1 and M2 models).

The findings of a significant increase in 7 out 36 Pi items between M1 and M2 warrant further consideration. In particular, taken singly they are the expression of several psychological aspects met during HFS training such as: i) the practitioners’ involvement in scenario performance in terms of familiarization with the training room, the awareness of what they need to do in scenario management (Pi4) and the consciousness of the efficacy of their DR management and resuscitation maneuvers (Pi13), ii) the intensity of practitioners’ involvement in scenario performance characterized by the absence of any psychological inhibition due to external evaluators and observers (Pi7, Pi25). In particular, they were not worried by or they do not care about audience judgment of their DR management (Pi34), and iii) the loss of time dimension that differed among practitioners in terms of duration quickly (Pi26) or loss (Pi35). The present patterns are in line with those detected in other activities at high risk for performance under stress such as intensivists, athletes and firefighters [25–29, 36–39, 42–45]. Altogether, the aforementioned results are reasonably supportive of the effectiveness of HFS training in terms of the fidelity of the scenarios to real life. The findings were furthermore corroborated by all practitioners, who during the debriefing phase, confirmed their feeling that after a few seconds from DR maneuvers starting they were supporting a newborn and not a simulator.

In the present study we also found that short dispositional flow scale items herein called SFSS significantly increased from M1 to M2. In particular, the combination of Pi items provided evidence that all the practitioners showed: i) a clear idea (SFSS2, SFSS4) of the stabilization and resuscitation procedures that needed to be performed in order to guarantee the best support to the newborn in DR, and ii) the highest level of focus on reaching the target (i.e. newborn safety). Results are in line with those detected in other activities at high risk for performance under stress such as intensivists, professional sportsmen and firefighters [37–49]. Altogether, it is reasonable to conclude that HFS training not only provides an enhancement of the awareness of the quality of NTS and TS performance but also the psychological involvement that is commonly met in real-assistance of high risk newborns.

Lastly, we recognize that the present study has several limitations. In particular: i) the evaluation of the impact of HFS training on clinical practice is today still the object of debate, ii) the need to correlate the level of psychological stress, during scenario performance, with new experimental video-computerized programs able to offer a qualitative and quantitative evaluation of DR management as for other NICU maneuvers [50]. In this light investigations over a wider study-population are required.

## Conclusions

In conclusion, the present results showing the high level of practitioners involvement during DR management simulations offer additional support to the usefulness of HFS as a trustworthy tool for improving the awareness of NTS and TS performance in neonatal care. The data open the way for further investigations aimed at the evaluation not only of individual but also of muldisciplinary team performances.

## Data Availability

The data will not be shared to respect the privacy of the participants.

## Abbreviations

HFS: High fidelity simulation
DR: Delivery room
FSS: Flow state scale
PPV: Positive pressure ventilation
TS: Technical skills
NTS: Non-technical skills
CRM: Crisis resource management
AHA: American Heart Association
AAP: Academy of Pediatrics
M1: Model 1
ERC: European Resuscitation Council
NRP: Neonatal Resuscitation Program
M2: Model 2
SFSS: Short flow state scale
